# Supplementary material for: Molecular mechanisms of urate transport by the native human URAT1 and its inhibition by anti-gout drugs
Source: Cell Discov. 2025 Apr 1;11:33. doi: 10.1038/s41421-025-00779-z (PMC11962085; doi:10.1038/s41421-025-00779-z)
Supplement: Supplementary file 1 — Supplementary Information [file 41421_2025_779_MOESM1_ESM.pdf]

Supplementary Materials for

Molecular mechanisms of urate transport by the native human URAT1 and its inhibition by anti-gout drugs

Canrong Wu *et al.*

✉Correspondence should be addressed to: H. Eric Xu (eric.xu@simm.ac.cn), Dehua Yang (dhyang@simm.ac.cn), Yi Jiang (yjiang@lglab.ac.cn), or Canrong Wu (wucanrong@simm.ac.cn)

This PDF file includes:

Figs. S1 to S10
Table S1 to S2

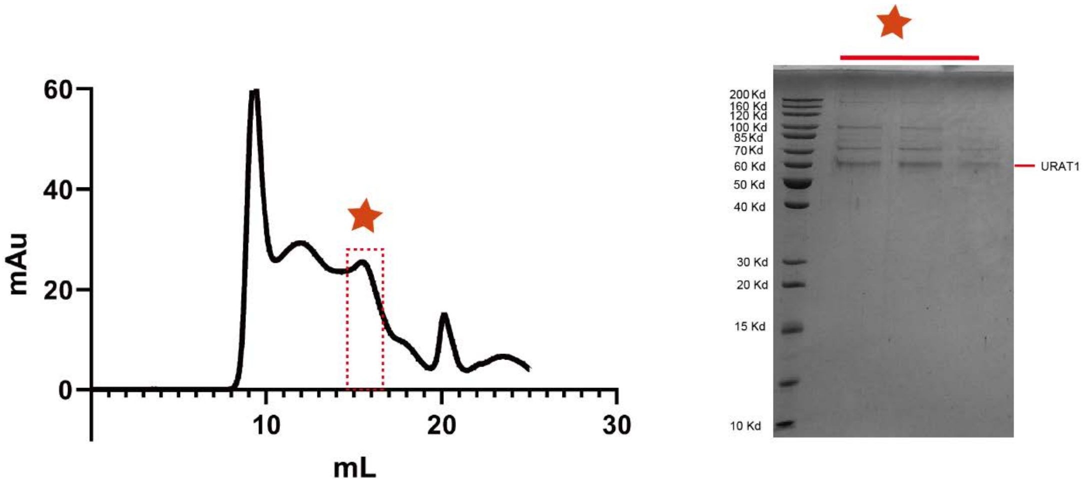

Supplementary Fig. 1. Representative size-exclusion chromatography and SDS-PAGE analysis of native hURAT1.

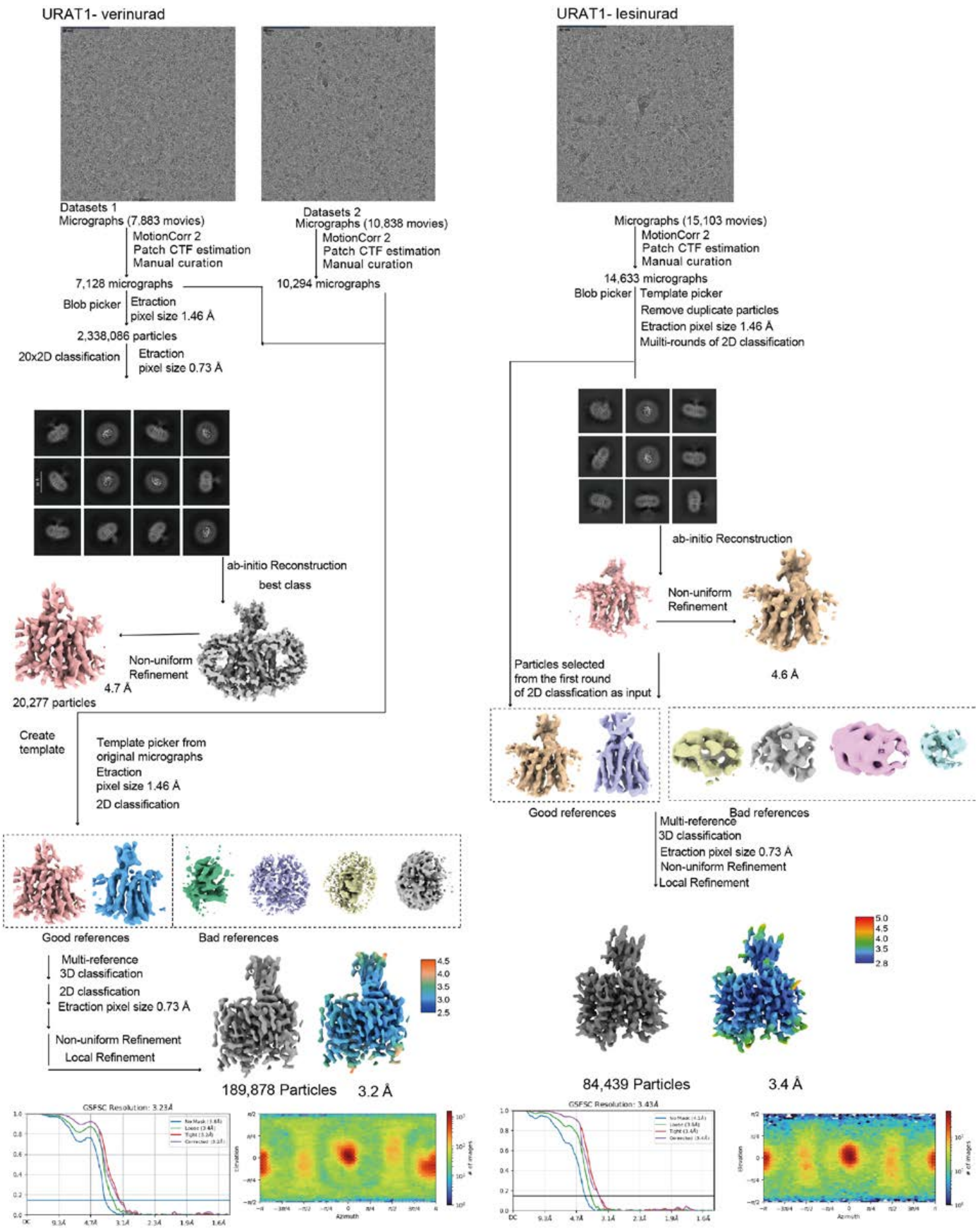

Supplementary Fig. 2. A flow-chart of the cryo-EM data process of the URAT1- verinurad and URAT1-lesinurad.

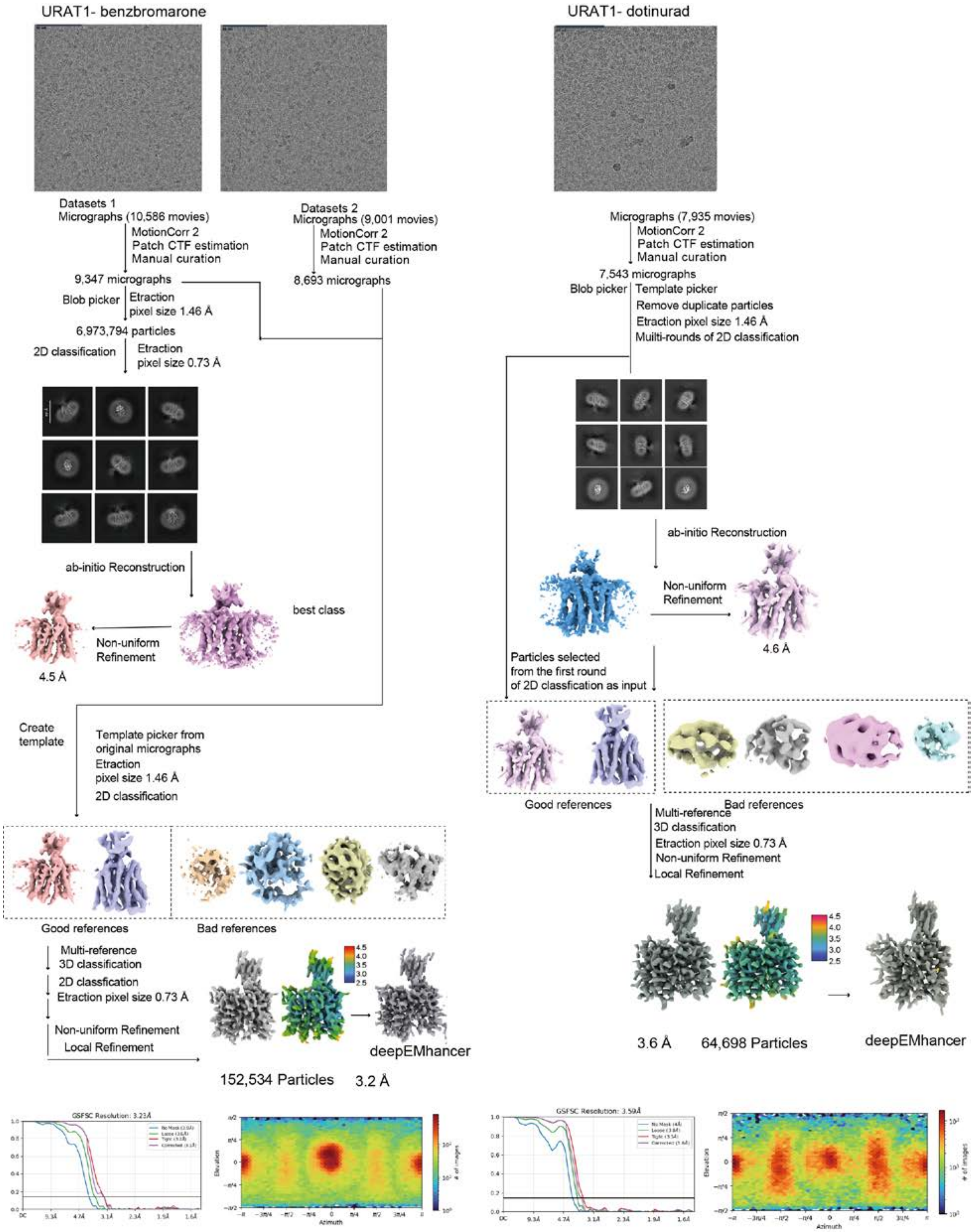

Supplementary Fig. 3. A flow-chart of the cryo-EM data process of the URAT1-benzbromarone and URAT1-dotinurad.

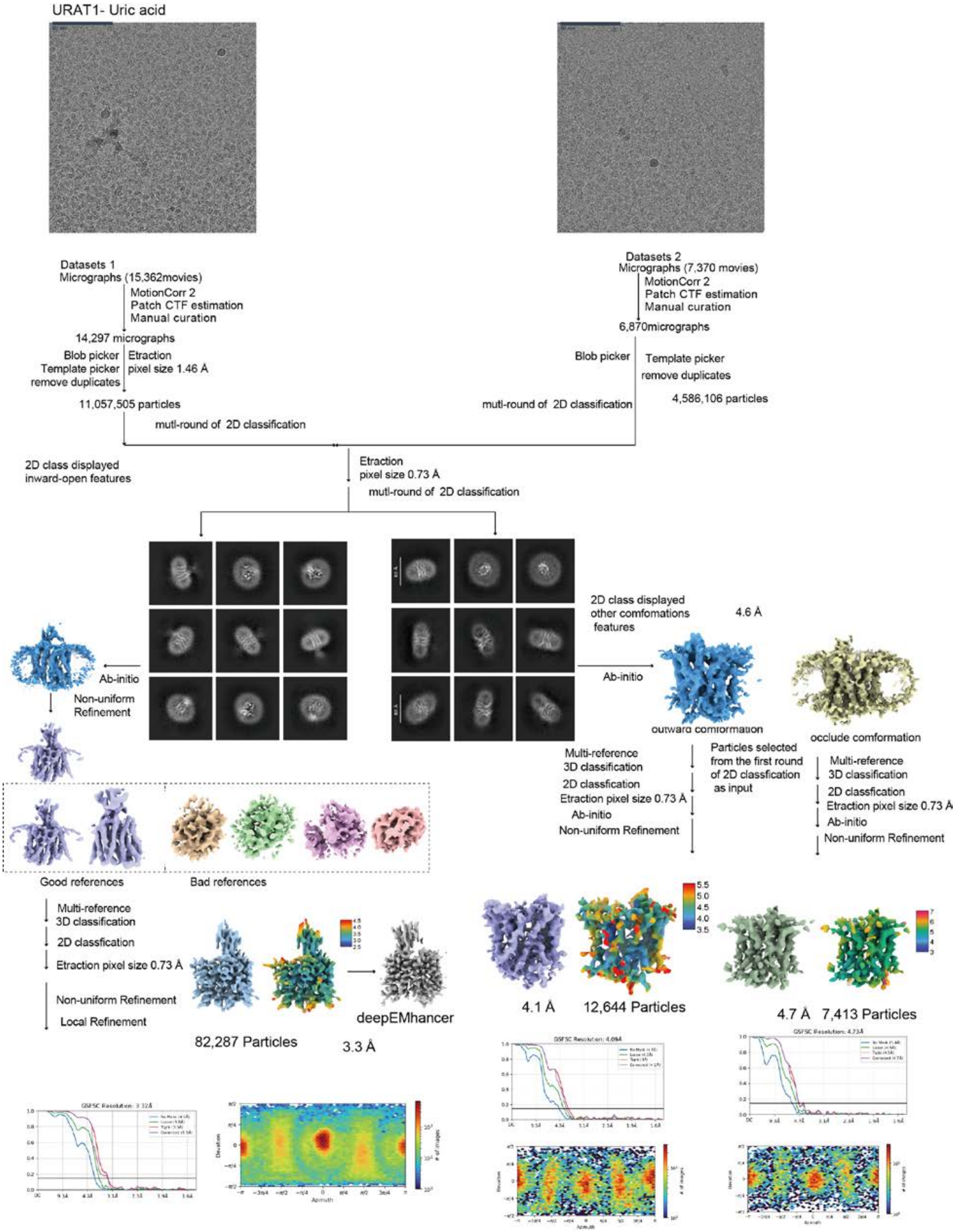

35
36 **Supplementary Fig. 4.** A flow-chart of the cryo-EM data process of the URAT1-urate.
37

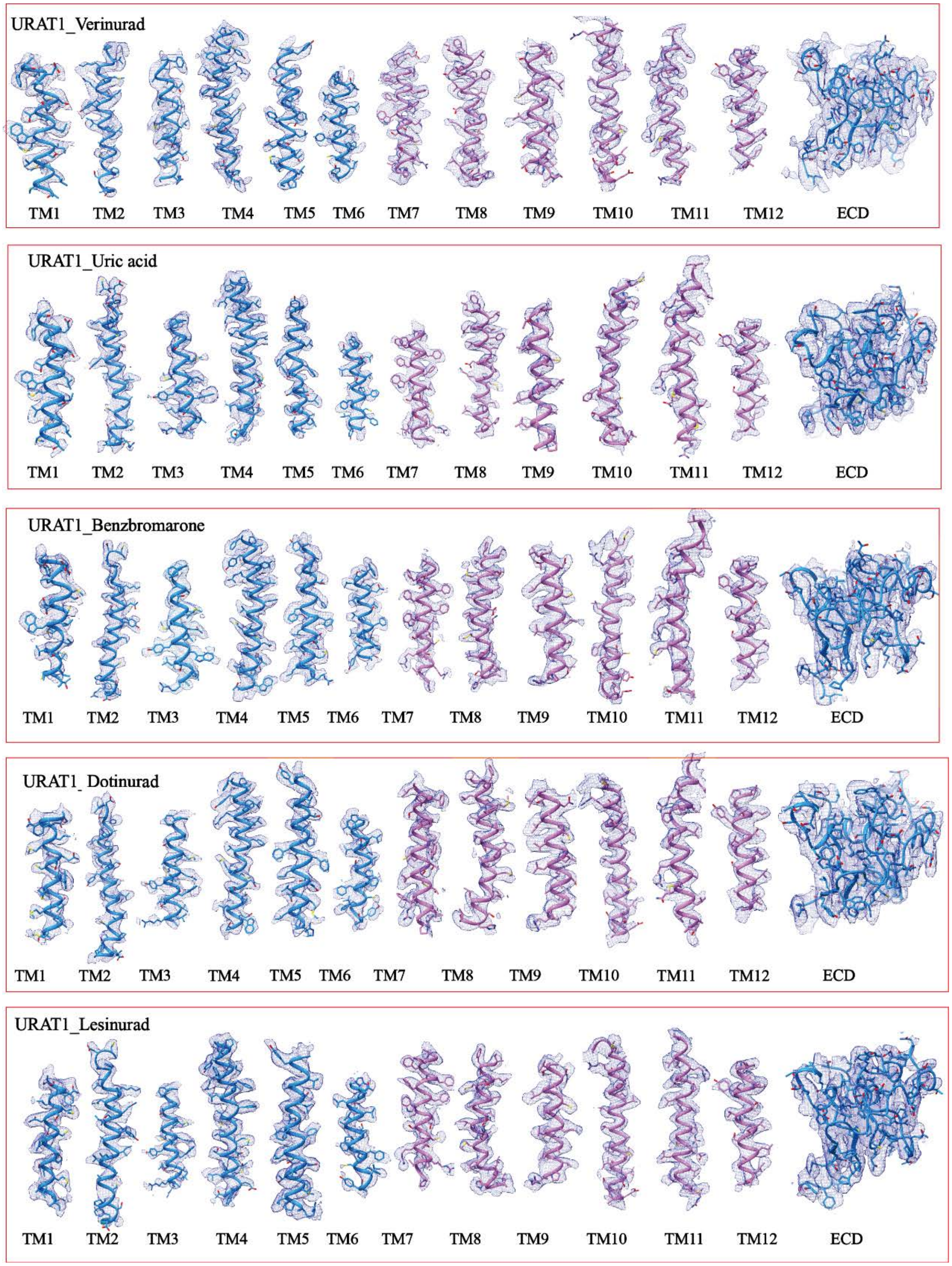

Supplementary Fig. 5. The cryo-EM density map of the representative regions of the five URAT1 structures.

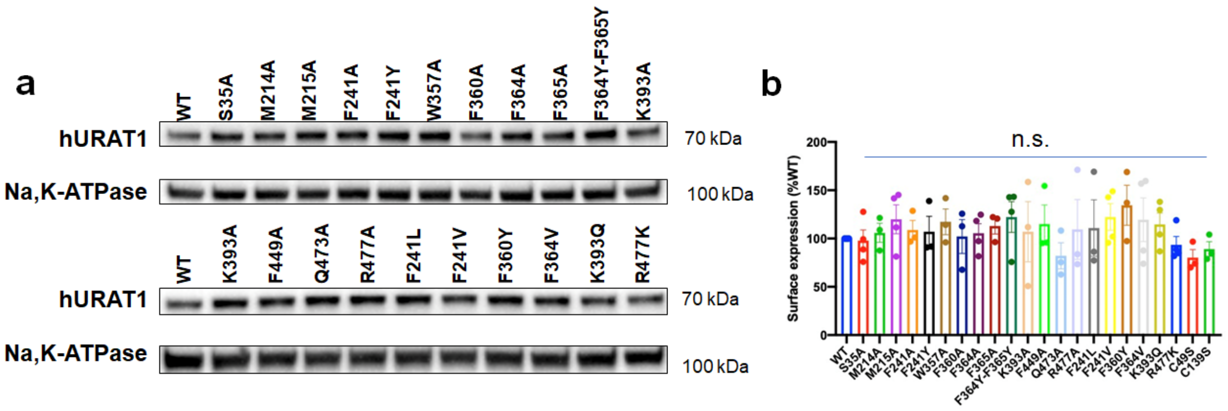

Supplementary Fig. 6. Surface Expression Levels Analyzed by Western Blotting for Control, WT, and Mutants. (**a**). Representative Western blot images showing the expression of WT and mutant proteins. Na+/K+-ATPase is used as a loading control for cell surface proteins. (**b**). Evaluated relative expression levels of each mutant compared to WT. Each point represents an independent experiment.

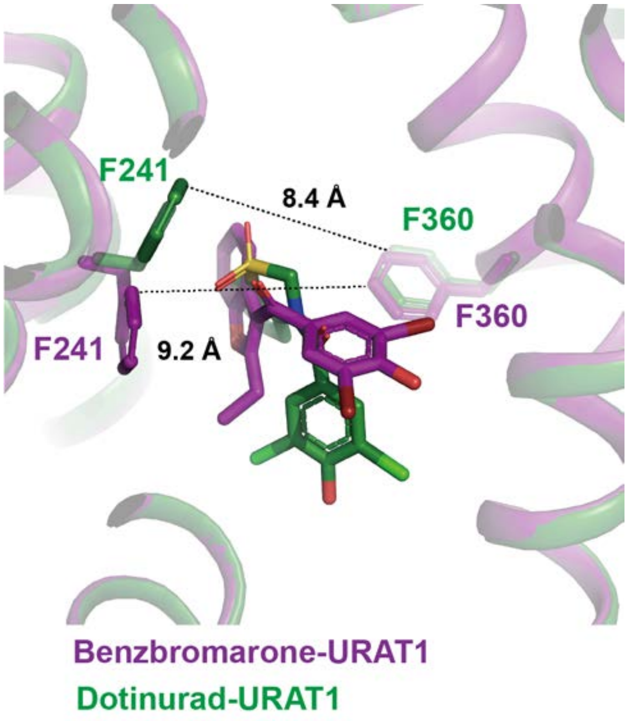

Supplementary Fig. 7. Different binding poses of benzbromarone and dotinurad in the URAT1 binding pocket.

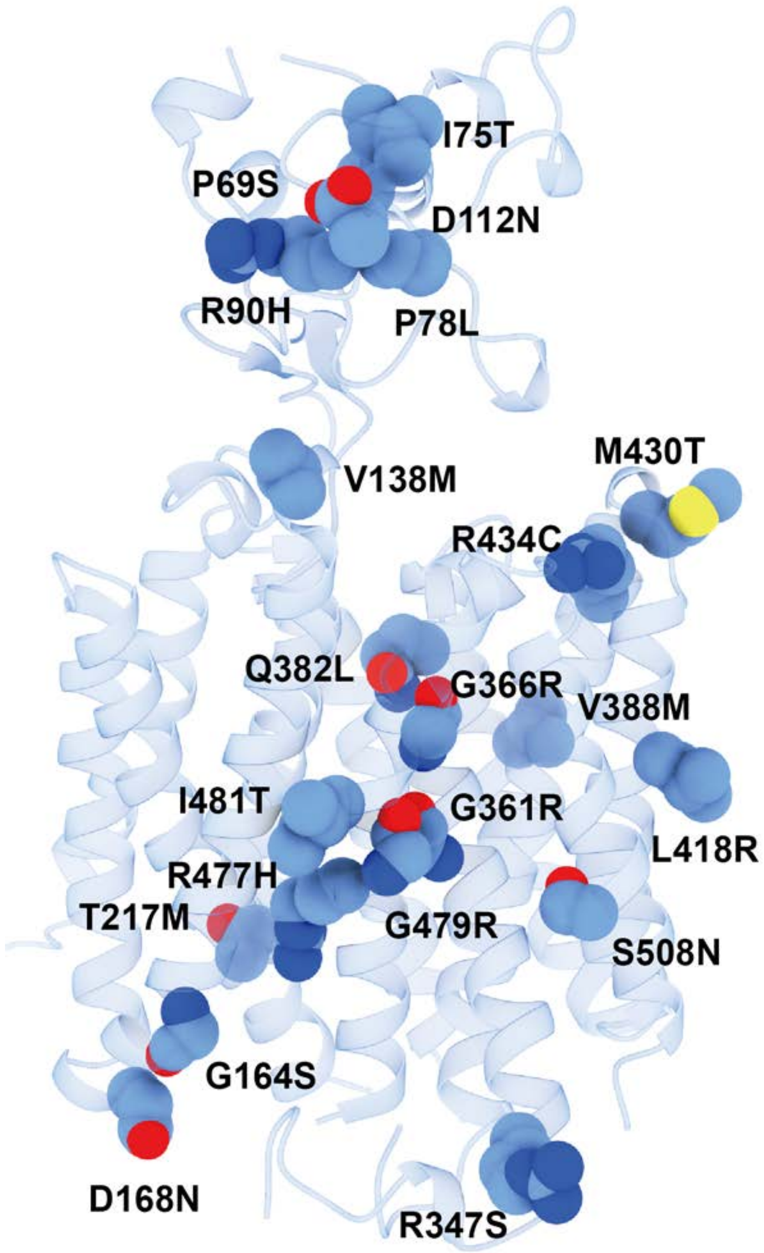

Supplementary Fig. 8. Mapping of natural URAT1 variants onto the structure of URAT1. Residues associated with natural human URAT1 variants are displayed as spheres.(data from https://www.ncbi.nlm.nih.gov/clinvar/)

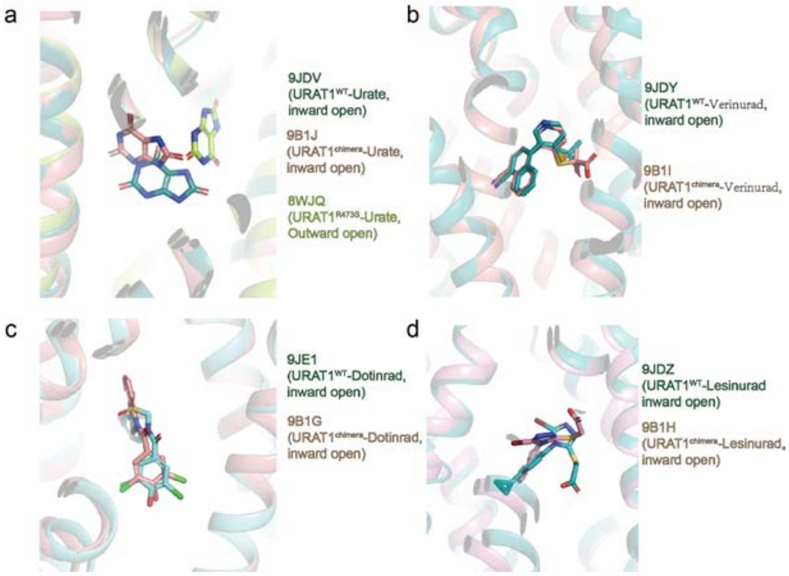

Supplementary Fig. 10. Superimposition of the URAT1^{WT} with URAT1^{R473S} and URAT1^{chimera} aligned at the ligand binding pocket. Structural comparison of the ligand binding pockets among URAT1 variants. Superposition of wild-type URAT1 (URAT1^{WT}, colored in teal) with the R473S mutant (URAT1^{R473S}, colored in limon) and chimeric construct (URAT1^{chimera}, colored in salmon), aligned at their respective ligand binding sites. Proteins are shown in cartoon representation, and ligands are displayed as sticks.

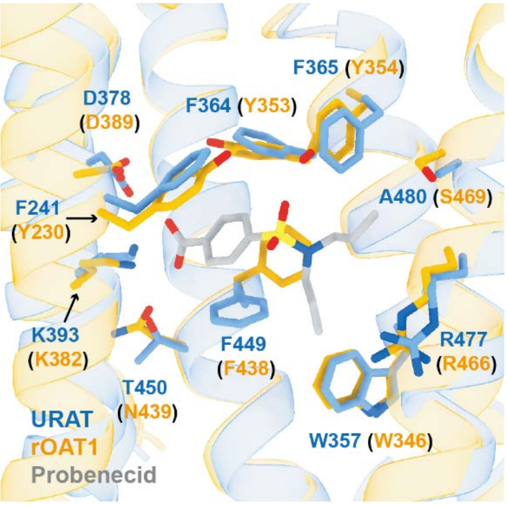

Supplementary Fig. 9. Superimposition of the hURAT1 with Probenecid bound rOAT1(PDB: 8SDZ) aligned at the ligand binding pocket.

Supplementary information, Table S1

Table S1. Cryo-EM data collection, model refinement and validation statistics.

	URAT1 verinurad	URAT1 dotinurad	URAT1 benzbromarone	URAT1 lesinurad	URAT1 Urate
**Data collection and processing**					
Detector	Falcon4	Falcon4	Falcon4	Falcon4	Falcon4
Magnification	165,000	165,000	165,000	165,000	165,000
Voltage (kV)	300	300	300	300	300
Electron exposure (e^{-}/Å^2)	50	50	50	50	50
Defocus range (μm)	-1.0~-3.0	-1.0~-3.0	-1.0~-3.0	-1.0~-3.0	-1.0~-3.0
Pixel size (Å)	0.73	0.73	0.73	0.73	0.73
Symmetry imposed	C1	C1	C1	C1	C1
Initial particle projections (no.)	11,742,200	5,792,635	10,776,212	8,668,180	15,643,611
Final particle projections (no.)	189,878	64,698	152,534	84,439	82,287
Map resolution (Å)	3.2	3.6	3.2	3.5	3.3
Map resolution range (Å)	2.5-4.5	2.5-4.5	2.5-4.5	2.8-4.5	2.5-4.5
FSC threshold	0.143	0.143	0.143	0.143	0.143
**Model Refinement**					
Refinement package	PHENIX-1.17.1-3660	PHENIX-1.17.1-3660	PHENIX-1.17.1-3660	PHENIX-1.17.1-3660	PHENIX-1.17.1-3660
Real or reciprocal space	Real space	Real space	Real space	Real space	
Model-Map CC (mask)	0.66	0.69	0.68	0.65	0.66
Model resolution (Å)	3.3	3.7	3.4	3.5	3.5
FSC threshold	0.143	0.143	0.143	0.143	0.143
B factors (Å^2, min/max/mean value)					
Protein residues	39.63/134.22/67.53	38.52/146.58/70.54	63.50/142.74/93.31	65.83/161.05/94.37	67.61/136.11/93.07
Ligands	56.88/56.88/56.88	63.23/63.23/63.23	102.29/102.29/102.29	97.38/97.38/97.38	90.05/90.05/90.05
**Model composition**					
Non-hydrogen atoms	3,324	3,321	3,322	3,463	3,324
Protein residues	435	435	435	440	435
R.m.s. deviations					
Bond lengths (Å)	0.001	0.002	0.002	0.002	0.001
Bond angles (°)	0.365	0.553	0.506	0.506	0.365
**Validation**					
MolProbity score	1.97	1.45	1.60	1.52	1.97
Clashscore	5.55	6.88	4.93	5.68	30.55
Rotamer outliers (%)	0.00	0.34	0.00	0.00	
Ramachandran plot					
Favored (%)	98.12	97.65	97.88	97.88	98.59
Allowed (%)	1.88	2.35	2.12	1.74	0.87
Disallowed (%)	0.00	0.00	0.00	0.00	0.00
**Data availability**					
EMDB entry	EMD-61401	EMD-61404	EMD-61403	EMD-61402	EMD-61399
PDB entry	9JDY	9JE1	9JE0	9JDZ	9JDV

Supplementary Table 2 Effects of hURAT1 mutations on the transporter activity of different drugs. The radiolabeled substrate-uptake assay of four compounds to the wild-type (WT) and mutated hURAT1 was performed in HEK293 cells with ^{14}C-urate. Data shown are means ± SEM of at least three independent biological replicates (n=3-8). The statistical analysis was performed by unpaired two-tailed t-test. (*P< 0.05, **P< 0.01, ***P< 0.001, *vs.* WT). N.A., not active.

**Mutations**	**pIC_{50} (Mean ± SEM)**			
	**Benzbromarone**	**Dotinurad**	**Lesinurad**	**Verinurad**
WT	6.71 ± 0.08	8.06 ± 0.06	4.64 ± 0.12	7.39 ± 0.07
F241L	7.28 ± 0.17 *	7.03 ± 0.13 ***	5.07 ± 0.13 *	7.35 ± 0.13
F360A	5.74 ± 0.12 ***	5.50 ± 0.36 ***	4.79 ± 0.19	7.25 ± 0.13
F364Y-F365Y	5.14 ± 0.13 ***	6.91 ± 0.18 ***	N.A.	3.61 ± 1.32 **
F449A	5.44 ± 0.19 ***	/	3.58 ± 0.25 **	6.90 ± 0.20 *
R477K	6.38 ± 0.10 *	7.64 ± 0.08 **	4.43 ± 0.09 *	6.29 ± 0.07 ***
